# *Plasmodium vivax* Malaria among Military Personnel, French Guiana, 1998–2008

**DOI:** 10.3201/eid1707.100009

**Published:** 2011-07

**Authors:** Benjamin Queyriaux, Gaëtan Texier, Lénaïck Ollivier, Laurent Galoisy-Guibal, Rémy Michel, Jean-Baptiste Meynard, Christophe Decam, Catherine Verret, Vincent Pommier de Santi, André Spiegel, Jean-Paul Boutin, René Migliani, Xavier Deparis

**Affiliations:** Author affiliations: Institut de Médicine Tropicale du Service de Santé des Armées, Marseille, France (B. Queyriaux, G. Texier, L. Ollivier, R. Michel, C. Decam, V. Pommier de Santi, J.-P. Boutin, X. Deparis);; Hôpital d’Instruction des Armées Desgenettes, Lyon, France (L. Galoisy-Guibal);; École du Val de Grâce, Paris, France (J.-B. Meynard, C. Verret, R. Migliani);; Institut Pasteur, Cayenne, France (A. Spiegel)

**Keywords:** malaria, Plasmodium vivax, French Guiana, military personnel, parasites, vector-borne infections, dispatch

## Abstract

We obtained health surveillance epidemiologic data on malaria among French military personnel deployed to French Guiana during 1998–2008. Incidence of *Plasmodium vivax* malaria increased and that of *P. falciparum* remained stable. This new epidemiologic situation has led to modification of malaria treatment for deployed military personnel.

French Guiana is a French Province located on the northern coast of South America that had 221,500 inhabitants in 2008 (*1*). Malaria is endemo-epidemic to the Amazon basin. Since 2000, the annual number of *Plasmodium falciparum* and *P*. *vivax* malaria cases in French Guiana has ranged from 3,500 to 4,500 (*2*). Approximately 3,000 French military personnel are deployed annually in French Guiana, and malaria occasionally affects their operational capabilities.

Only military personnel on duty in the Amazon basin are required to take malaria chemoprophylaxis; personnel deployed in coast regions are not. Until February 2001, the chemoprophylaxis regimen consisted of chloroquine (100 mg/d) and proguanil (200 mg/d). During March 2001–October 2003, mefloquine (250 mg/wk) was used. Since November, 2003 malaria chemoprophylaxis has been doxycycline (100 mg/d), which is initiated on arrival in the Amazon basin. All chemoprophylaxis is continued until 4 weeks after departure. Because of the absence of marketing authorization as chemoprophylaxis by the French Medicines Agency, primaquine was not used until recently. Other individual and collective protective measures did not change during 1998–2008.

Despite the availability of chemoprophylaxis, since 2003, several malaria outbreaks have been identified after operations against illegal mining in the Amazon basin (*3**,**4*). The purpose of those studies was to describe outbreaks and determine factors related to malaria cases. We report French military health surveillance epidemiologic data on malaria among military personnel deployed to French Guiana during 1998–2008.

## The Study

Epidemiologic malaria surveillance in French Armed Forces consists of continuous and systematic collection, analysis, interpretation, and feedback of epidemiologic data from all military physicians (Technical Appendix). Malaria is defined as any pathologic event or symptom associated with confirmed parasitologic evidence (*Plasmodium* spp. on a blood smear, a positive quantitative buffy coat malaria diagnosis test result, or a positive malaria rapid diagnosis test result) contracted in French Guiana. A case occurring in a person during or after a stay in French Guiana without a subsequent stay in another malaria-endemic area was assumed to be contracted in French Guiana. Each malaria attack was considered a separate case. Equal information was available for the entire 11-year study period. Data from weekly reports and malaria-specific forms were used for analysis.

Indicators are expressed as annual incidence and annual incidence rate. The denominator of the annual incidence rate is the average number of military personnel at risk for malaria during a given year.

Statistical analysis was performed by using Epi Info 6.04dfr (Centers for Disease Control and Prevention, Atlanta, GA, USA). Comparisons over time were made by using the χ^2^ test for trend and between groups by using the Kruskal-Wallis test. A p value <0.05 was considered significant.

The incidence rate for malaria cases among French military personnel deployed to French Guiana has increased since 1998 (p<0.001). *P*. *falciparum* incidence has remained stable (p = 0.10), and *P*. *vivax* incidence has increased (p<0.001) (Figure). In 2007 and 2008, French military personnel in French Guiana represented only 23.0% and 22.2% of those deployed to malaria-endemic regions. However, most reported malaria cases were contracted in this region (50.0% and 62.9%, respectively, of all cases). *P*. *vivax* was responsible for most malaria attacks reported in French Guiana (Table). The proportion of malaria attacks caused by *P*. *vivax* increased from 44% to 84% (p<0.001) during the study period.

**Figure:**
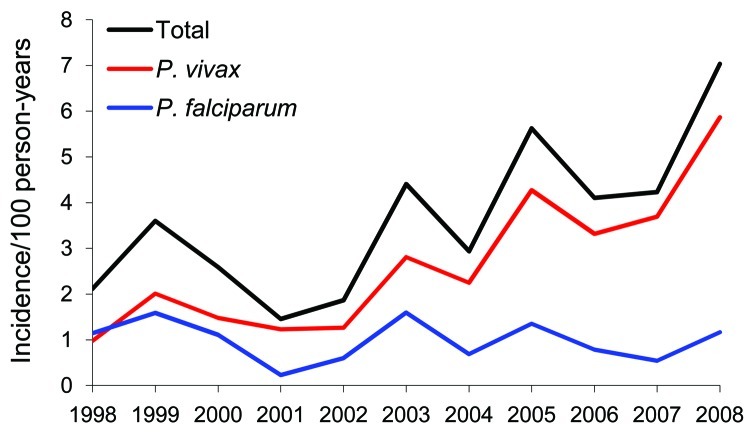
Incidence of malaria cases among French Armed Forces, by *Plasmodium* species, French Guiana, 1998–2008.

**Table:** Cases of *Plasmodium* spp. malaria among French Armed Forces, French Guiana, 1998–2008*

Year	Species				
	*P. falciparum*	*P. vivax*	*P. malariae*	*P. ovale*	Unknown
1998	41	35	0	1	3
1999	56	71	1	0	4
2000	36	48	0	2	5
2001	7	38	0	3	1
2002	19	40	1	2	0
2003	54	95	0	0	0
2004	22	72	1	3	0
2005	50	158	3	1	0
2006	29	123	0	2	0
2007	20	137	1	0	0
2008	44	221	0	0	0

*Cases of malaria caused by 2 parasites (co-infections) were included for each involved species.

In 2008, among the 264 reported cases, 221 were temporarily unavailable for duty. Median lost work days were lower for attacks of *P*. *vivax* malaria than for *P*. *falciparum* malaria (5 days/attack, interquartile range 4–7 days/attack vs. 7 days/attack, interquartile range 5–10 days/attack; p = 0.006)

Among 264 malaria cases contracted in French Guiana, 39.4% were in persons who had reported >1 malaria attack in the previous 6 months. Among the 221 *P*. *vivax* malaria cases, 45.5% were in persons who had already reported >1 *P*. *vivax* malaria attack in the previous 6 months. In 2008, among those required to take chemoprophylaxis (i.e., during a mission to the Amazon basin and 28 days after the mission), 45.0% admitted not taking their chemoprophylaxis within 8 days before onset of symptoms.

## Conclusions

*P*. *vivax* malaria attacks have resulted in a substantial number of lost work days and have adversely affected operational readiness of military personnel. Despite availability of appropriate chemoprophylaxis, since 1998, French Armed Forces have been affected by an increase in incidence of *P*. *vivax* malaria. Several causes of this increase have been hypothesized.

First, epidemiologic trends for all-cause malaria in French Guiana and overall reported malaria incidence has not changed substantially since the end of the 20th century: ≈4 000 cases were reported annually during the 1990s (*5*) and 3,500–4,500 were reported during 2008 (*2*). However, the proportion of *P*. *vivax* malaria has increased from 20% of cases in the 1990s (*6*) to 56.1% during September 2003–February 2004 among patients at the Cayenne Public Hospital (*7*). Furthermore, 70% of malaria cases diagnosed in 2006 among French travelers returning from French Guiana were caused by *P*. *vivax* (*8*). One explanation for this parasitologic evolution may involve immigration from Brazil and Suriname to illegal gold-mining areas in the Amazon basin of French Guiana (*2**,**6*). These immigrating populations brought *P*. *vivax* from high-prevalence regions to an area where an efficient vector for malaria, *Anopheles darlingi* mosquitoes, was present (*9*). Changes in weather patterns and regional infrastructure could also explain this increase.

Second, military missions have intensified. In 2008, a police and military operation to reduce illegal gold-panning activities in the Amazon basin occurred in French Guiana. This operation might explain the 2008 peak in the incidence rate. Since 2002, these operations have resulted in several outbreaks among forces in French Guiana, especially in 2003 and 2005 (*2**,**3*).

Third, deficiencies have occurred in implementing individual and collective protective measures. These military operations were conducted by personnel from French Guiana or France, few had any rainforest experience. Despite extensive training, instructions were clearly not followed, as demonstrated by a 45% noncompliance rate for chemoprophylaxis.

*P*. *vivax* has accounted for >80% of reported malaria cases in French Guiana for the past 3 years. In addition, relapses of *P*. *vivax* malaria occur in the absence of radical treatment. In 2008, 45.5% of persons with *P*. *vivax* malaria had already reported >1 *P*. *vivax* malaria attack in the past 6 months. Although the *P*. *vivax* malaria mortality rate is low, the effect of *P*. *vivax* malaria on force operational readiness is high because relapses decrease the availability of military personnel. In addition, *P*. *vivax* malaria can be severe, despite its reputation as a mild form of malaria (*10*). Since 2009, to reduce the number of relapses, a French Ministry of Defense circular has recommended treatment with primaquine for 2 or 3 weeks after a first attack of *P*. *vivax* malaria. Studies of the use of primaquine chemoprophylaxis are ongoing (*11**–**13*).

In conclusion, the incidence of *P*. *vivax* malaria is increasing in French Guiana, especially in French Armed Forces. The incidence of *P*. *falciparum* malaria remains stable. This new epidemiologic finding can affect the level of individual health and operational capabilities. Performance of vector evaluation studies and control in the regions could be another possible intervention.

## Supplementary Material

Technical Appendix The French Armed Forces Epidemiologic Surveillance System.
