# Supplementary material for: Plasmodium vivax Malaria among Military Personnel, French Guiana, 1998–2008
Source: Emerg Infect Dis. 2011 Jul;17(7):1280–2. doi: 10.3201/eid1707.100009 (PMC3381416; doi:10.3201/eid1707.100009)
Supplement: Technical Appendix — The French Armed Forces Epidemiologic Surveillance System. [file 10-0009-Techapp.pdf]

Article DOI: 10.3201/eid1707.100009

Plasmodium vivax Malaria among Military Personnel, French Guiana, 1998–2008

Technical Appendix

The French Armed Forces Epidemiologic Surveillance System

Epidemiologic surveillance in French Armed Forces comprises continuous and systematic collection, analysis, interpretation, and feedback of epidemiologic data from all French military physicians worldwide. Sixty-two medical events, e.g., tuberculosis, measles, or amibiasis cases; immunization adverse effects; and suicide or death, are followed. These events have a public health interest and a military interest, or their reporting is mandated by French civilian health authorities.

Each military physician reports weekly to the French Armed Forces epidemiologic and public health departments the number of cases for each event and the exposed population. The studied population includes any French military personnel, regardless of status, rank, location, or branch of service. Each event has strict notice criteria to standardize reporting. The main goals of this weekly reporting are to follow trends and to detect outbreaks. Moreover, in case of high epidemiologic risk (e.g., meningitides, cholera), physicians must immediately report case(s) to permit a quick epidemiologic intervention.

For 51 events (e.g., malaria, tuberculosis), physicians are required to complete a specific form for each reported case. This form, which is anonymous, gathers information about patient demographic characteristics, circumstances, clinical features, implemented treatment, and evolution of the illness. A yearly analysis of these forms provides information about epidemiologic features, long-term trends, and efficiency of public health policies.

Results of weekly and yearly analyses are sent to the French Surgeon General, the French Forces Headquarters, the French civilian health authorities, and all military physicians (feedback). Interesting and original results are published in medical journals.

A new syndromic near–real-time epidemiologic surveillance system named ASTER (alerte et surveillance en temps réel) is being developed to enhance the French epidemiologic capacity to detect possible outbreaks as soon as possible. This system will complete the weekly epidemiologic system.
